# Wheat Germ Fermentation with *Saccharomyces cerevisiae* and *Lactobacillus plantarum*: Process Optimization for Enhanced Composition and Antioxidant Properties In Vitro

**DOI:** 10.3390/foods11081125

**Published:** 2022-04-14

**Authors:** Elnaz Bayat, Marzieh Moosavi-Nasab, Mahboubeh Fazaeli, Marjan Majdinasab, Armin Mirzapour-Kouhdasht, Marco Garcia-Vaquero

**Affiliations:** 1 Department of Food Science and Technology, School of Agriculture, Shiraz University, Shiraz 71946-84471, Iran; bayatasl211@gmail.com (E.B.); fazaeli@shirazu.ac.ir (M.F.); majdinasab@shirazu.ac.ir (M.M.); 2 Seafood Processing Research Group, School of Agriculture, Shiraz University, Shiraz 71946-84471, Iran; 3 School of Agriculture and Food Science, University College Dublin, D04 HK50 Dublin, Ireland; armin.mirzapourkouhdasht@ucd.ie

**Keywords:** gamma-aminobutyric acid, GABA, quinone, peptides, *Lactobacillus plantarum*, *Saccharomyces cerevisiae*, wheat germ, antioxidant

## Abstract

Wheat germ, a by-product of the flour milling industry, is currently commercialized mainly for animal feed applications. This study aims to explore and optimize the process of wheat germ fermentation to achieve products with enhanced nutritional composition and biological properties and further characterize the fermented products generated using these optimum conditions. The type of microorganism (*Saccharomyces cerevisiae* 5022 (yeast) and *Lactobacillus plantarum* strain 299v (bacteria)), pH (4.5, 6, and 7.5) and fermentation time (24, 48, and 72 h) were optimized using response surface methodology (RSM) aiming to achieve fermented products with high total phenol content (TPC), dimethoxy benzoquinone (DMBQ) and antioxidant activities. Optimum fermentation conditions were achieved using *L. plantarum*, pH 6, 48 h, generating extracts containing TPC (3.33 mg gallic acid equivalents/g), DMBQ (0.56 mg DMBQ/g), and DPPH radical scavenging (86.49%). These optimally fermented products had higher peptide concentrations (607 μg/mL), gamma-aminobutyric acid (GABA) (19,983.88 mg/kg) contents compared to non-fermented or yeast-fermented products. These findings highlight the influence of fermentation conditions of wheat germ and the promising industrial application of wheat germ fermentation for developing food products with enhanced biological properties promising for their commercialization as functional foods.

## 1. Introduction

Wheat is one of the main staple foods in several countries around the globe, serving as an essential commodity to over one-third of the world’s population, contributing more than any other crop to the caloric intake of this population [1]. Wheat grain constituents can be divided into endosperm (80–85%), bran (13–17%), and germ (2–3%), the latter one currently being considered a by-product of the flour milling industry. The presence of wheat germ negatively affects the technological and quality attributes of flour and the stability of dough when the flour is used in bread-making processes [2,3]. Wheat germ has been described as a source of macronutrients (proteins and peptides, carbohydrates and lipids, as well as other minor compounds with proven health benefits when used as functional foods, such as tocopherols, phytosterols, carotenoids, thiamin, riboflavin, niacin, phenolics, saponins, flavonoids, γ-aminobutyric acid (GABA), and quinones [3]. Furthermore, wheat germ is also known to possess a well-balanced amino acid profile and has relatively rich contents of essential amino acids, especially lysine, methionine, and threonine [4]. Despite all these nutritional benefits, some anti-nutritional factors (i.e., phytic acid, raffinose, and agglutinin) have also been described in wheat germ. Other disadvantages of this product include its short shelf-life, mainly attributed to the high levels of unsaturated fatty acids and lipid-metabolizing enzymes that induce a rapid lipid degradation and rancidity, limiting its inclusion in food formulations [5].

Due to these limitations, the 25 million tons of wheat germ currently produced by the milling industries [2] are currently underutilized, as the main market of this product currently focuses on low-value commercial applications, mainly as dietary supplements in animal feed formulations [6].

Thermal treatments have been explored to stabilize and improve the wheat germ’s shelf-life, although these techniques reduced the nutritional value of these ingredients [7]. On the other hand, other biological processes, such as fermentation, have also been explored as a promising strategy to inactive anti-nutritional factors present in wheat germ as well as to improve its nutritional value [2,8].

Moreover, several studies emphasized that cereal fermentation with yeast or lactic acid bacteria (LAB) as a promising strategy to increase the levels of bioactive compounds and biological properties of cereals and cereal by-products. Thus, the enzymatic action of microorganisms during fermentation can induce the release of phenolic compounds by the breakage of the bonds binding phenolics with other constituents, also inducing an improvement of the antioxidant activities of the fermented products [9] as well as generating and releasing peptides that may also contribute to enhancing the biological properties of the fermented cereals [10]. LAB is widely used in food fermentation, *L. plantarum* being the most frequently used species to ferment food products of plant origin [11] and also recently in wheat germ to enhance the release of bioactive compounds [2]. *S. cerevisiae* was also used to ferment wheat germ, enriching the product with bioactive compounds with antioxidant and anticarcinogenic biological properties. Moreover, a commercially available wheat germ extract commercialized as Avemar^®^ is currently used as a nutritional supplement with anticancer properties that are likely to be attributed to their contents of quinones, such as 2,6-dimethoxy-1,4-benzoquinone (DMBQ), with known antimicrobial and immunostimulatory effects [12,13].

When using any fermentation process, the type of microorganism and the parameters affecting their growth and enzymatic activities, mainly pH, temperature, and the time of fermentation, will determine the final quality of the fermented products. [14]. However, to our knowledge, no information is available on optimizing the fermentation process of wheat germ using *L. plantarum* and *S. cerevisiae* to ensure obtaining a fermented wheat germ of the highest biological and nutritional qualities.

The aim of this study is (1) to explore and determine the optimum fermentation conditions of wheat germ, focusing on pH (4.5, 6, and 7.5), fermentation time (24, 48, and 72 h) and type of microorganism (*S. cerevisiae* PTCC 5022 or *L. plantarum* PTCC 299V) to achieve products with enhanced total phenol content (TPC), dimethoxy benzoquinone (DMBQ), and antioxidant activities (1,1-diphenyl-2-picryl-hydrazyl (DPPH) radical scavenging activity); and (2) to analyze further the additional benefits of the fermented products generated using optimum fermentation conditions by analyzing the presence of other bioactive compounds (peptide and GABA contents) in these fermented products.

## 2. Materials and Methods

### 2.1. Biological Materials

Wheat germ (Shiraz wheat cultivar) was obtained from Khousheh Fars Flour Milling Plant (Shiraz, Iran) and stored at −18 °C to avoid lipid oxidation and other undesirable changes in the biological material prior to further processing. YGC (Yeast Extract Glucose Chloramphenicol) medium and MRS (Modified deMan, Rogosa, and Sharpe) culture media were purchased from Merck Co. (Darmstadt, Germany). Lyophilized *S. cerevisiae* (PTCC 5022) and *L. plantarum* (PTCC 299V) cultures were purchased from the Iran Organization for Research and Technology’s culture collection (Tehran, Iran). YGC culture medium was incubated in a shaking incubator at 120 rpm and 28 °C for 48 h. Thereafter, an aliquot of 100 μL of *L. plantarum* was added to 5 mL of the MRS culture medium and incubated further at 37 °C for 24 h. Before the inoculation, to efficiently activate the MRS medium, 2 mL of this culture medium containing the *L. plantarum* was added to 50 mL of the MRS media and incubated under identical conditions for 48 h.

### 2.2. Chemical Reagents

Methanol and ethanol of HPLC grade, Folin-Ciocalteu reagent, gallic acid, 1,1-diphenyl-2-picrylhydrazyl (DPPH), 2,6-dimethoxy-1,4-benzoquinone (DMBQ), and γ-aminobutyric acid (GABA), were purchased from Sigma Chemical Co. (St. Louis, MO, USA). All other chemicals and reagents were of analytical grade and were purchased from Sinopharm Chemical Reagent Co., Ltd. (Shanghai, China).

### 2.3. Wheat Germ Fermentation

10 g of the wheat germ was mixed into 200 mL of sodium phosphate buffer solution (0.05 M). Bacterial and yeast cells were then separated from the culture medium by centrifugation (6000× *g*, 5 min at room temperature). The harvested cells were then washed with sterile phosphate buffer multiple times, resuspended in water to achieve a cell population of 10^8^ CFU/mL, and homogenized using a vortex unit. The yeast and bacterial cells were fermented at 28 °C and 37 °C, respectively, with variable fermentation times (24, 48, and 72 h) and pH levels (4.5, 6.0, and 7.5) required for the process of optimization as described in detail in Section 2.6. Upon the completion of each fermentation process, the samples were freeze-dried (Christ ALPHA 1–2 LD plus, Osterode am Harz, Germany) and preserved at −20 °C for further chemical analyses.

### 2.4. Chemical Analyses

All the chemical analyses were performed in triplicate.

#### 2.4.1. Proximate Composition Analyses

The moisture, ash, fat, and protein contents of the samples were determined according to the official methods of analysis AACC 44-15, 08-12, 30-10, and 46-12, respectively (AACC, 2001). For moisture content determination, samples (1 g) were weighed in pre-weighed Petri dishes and oven-dried at 105 ± 2 °C for 5 h. The samples were subsequently cooled to room temperature in a desiccator and weighed. The difference in sample weight before and after oven-drying represents the moisture content of the samples. For ash content determination, samples (1 g) were weighed in pre-weighed crucibles and placed in a muffle furnace at 550 °C for 4 h. The crucibles were cooled down in desiccators and re-weighed. The difference in sample weight before and after the process is the ash content. For protein content, samples (1 g) were digested with 50 mL sulphuric acid in the presence of catalyst tablets. The digestion process consists of heating the samples for 30 min at 220 °C followed by 120 min at 420 °C. Subsequently, samples were cooled down to room temperature in a desiccator and were distilled using an Auto-Kjeldahl apparatus (BUCHI Labortechnik AG, Flawil, Switzerland). The fat contents were determined using the Soxhlet extraction method using 1 g of samples and 100 mL hexane for 6 h at 68 °C.

#### 2.4.2. Total Phenolic Content (TPC) Analysis

TPC was measured using the method adapted from Liu, Chen, Shao, Wang, and Zhan [10]. Briefly, the Folin–Ciocalteu phenol reagent (2N) was diluted ten times using distilled water. 0.1 mL of sample or standard (gallic acid, 0.1–10 mg/mL) were mixed with 0.75 mL of the diluted Folin–Ciocalteu phenol reagent and the mixtures were incubated at 20° C for 10 min. Following this incubation, 0.75 mL sodium carbonate solution (2% *w*/*v*) were added to each mixture, vortexed and incubated in dark conditions for 45 min. The absorbance of the mixtures was read at 765 nm using a spectrophotometer (UV-1650PC; Shimadzu Corp., Kyoto, Japan). The TPC results were expressed as mg gallic acid equivalents (GAE) per g of freeze-dried sample (mg GAE/g).

#### 2.4.3. DMBQ Analysis

DMBQ contents were measured using an HPLC system equipped with a quaternary pump (Knauer pump 1000, Berlin, Germany). The samples were prepared for DMBQ analysis following the protocol, as described by Zheng et al. [15]. Briefly, 10 g of samples were dissolved in 250 mL of distilled water and extracted three times using 200 mL of chloroform. The chloroform layers were collected, washed three times with distilled water, and dried over anhydrous sodium sulfate. The filtrates were evaporated using a vacuum evaporator (Rotavapor RII, BUCHI, Flawil, Switzerland) at 30 °C. The dried samples were re-dissolved in the mobile phase (20% acetonitrile and 80% water *v*/*v*) and filtered through 0.45 μm filters before their injection into the HPLC system. The HPLC system was equipped with a quaternary pump (Knauer pump 1000, Berlin, Germany), a UV detector (245 nm) and a C-18 column (5 μm, 250 × 4.6 mm; Nucleodur C18 pyramid 250/4.6, Macherey-Nagel, Düren, Germany). The mobile phase consisted of 20% acetonitrile−80% water (*v*/*v*) mixture at a flow rate of 0.5 mL/min and a temperature of 25 °C. Peaks were detected based on retention time, and DMBQ concentrations were determined by comparison with the standard (DMBQ 97%, ACROS Organics). All the measurements were conducted in triplicate, and the results were reported as mg/g DMBQ per g of the freeze-dried sample [15].

#### 2.4.4. Peptide Content Analysis

Peptide content analysis was performed following the protocol as described by Liu, Chen, Shao, Wang and Zhan [10] with slight modifications. Briefly, 0.25 mL of freeze-dried wheat germ were mixed with 2 mL of 0.2 M sodium phosphate buffer (pH 8.2) followed by 2 mL trinitrobenzenesulfonic acid 0.1 (*v*/*v*) (TNBS). The mixtures were incubated at 60 °C for 1 h under dark conditions and the reaction was stopped by adding 4 mL of HCl (0.1 M). The absorbance of each sample was recorded at 340 nm and the peptide content of each sample was quantitatively determined using L-leucine amino acid as the standard at concentrations ranging from 0 to 1.2 mg/mL [4].

#### 2.4.5. γ-Aminobutyric Acid (GABA) Analysis

GABA content of the samples was determined according to the method described by Donkor et al. [16] with modification. 0.25 g of wheat germ were mixed with 1 mL of 70% (*v*/*v*) ethanol, homogenized for 10 min in a vortex and centrifuged (10,000 rpm, 10 min, 4 °C). This process was repeated twice, the supernatants were pooled and the ethanol evaporated at 40 °C. The samples were re-dissolved in 1 mL of distilled water and cleaned through a 0.45 μm filter. The GABA content was determined by injecting 20 μL of the extract into the same HPLC system (HPLC column Nucleodur C18 Pyramaid 125 × 3 mm, 5 µm) equipped with a refractive index (RI) detector (Wyatt, Optilab rEX) and column (1000 Kanaber, Germany). The temperature of the column was set to 25 °C and HPLC grade water was used in column stationary phase at a flow rate of 0.6 mL/min.

### 2.5. DPPH Radical Scavenging Activity Determination

DPPH radical scavenging activity assays were performed in triplicate following the protocol, as described by by Liu, Chen, Shao, Wang, and Zhan [10] with some modifications. Briefly, 2 mL of wheat germ extract were diluted with 100 mL 90% methanol aqueous solution. 2 mL of extract were mixed with 1 mL of DPPH stock solution (4 mg per 100 mL of solvent 90% methanol) and the mixtures were incubated in the dark for 45 min. The absorbance of the samples was read at 517 nm. A methanolic solution containing all reagents without the addition of a test compound was used as a control. The DPPH radical scavenging activity of the samples was calculated using the following equation:

(1) $$\mathrm{DPPH}\;(\%)=\frac{A_{C}-A_{S}}{A_{C}}\times 100$$

where A_C_ is the absorbance of the control, and A_S_ represents the absorbance of the samples.

### 2.6. Experimental Design for Optimization

Response surface methodology (RSM) was used to optimize the fermentation of wheat germ using the software Design Expert (v 12.0, Stat-Ease US). The optimization of the fermentation process focused on the parameters pH (X_1_), time (X_2_), and type of microorganism (X_3_) to achieve products with maximum TPC and DMBQ contents as well as maximum DPPH radical scavenging activities. The type of microorganism are categorical factors introduced in the design as level 1 = bacteria and level 2 = yeast, while the independent variables were coded as X_1_ (−1 = 4.5, 0 = 6, +1 = 7.5) and X_2_ (−1 = 24, 0 = 48, +1 = 72).

Twenty-six experimental runs were performed following a central composite design. The different combinations of the process parameters were studied and the main responses achieved in each fermentation run are summarized in Table 1. The correlation between independent and dependent variables was explained through the second-order polynomial model outlined in the following equation.

(2) $$Y=\beta_{0}+\sum_{i=1}^{k}\beta_{i}X_{i}+\sum_{i=1}^{k}\beta_{\mathrm{ii}}X_{i}^{2}+\sum_{i=1}^{k-1}\sum_{j=i+1}^{k}\beta_{\mathrm{ij}}X_{i}X_{j}$$

where Y stands for a predicted response (TPC, DMBQ, and DPPH radical scavenging activity); β_0_, β_i_, β_ii_ and β_ij_ represent regression coefficients; and X_i_ and X_j_ are the coded independent factors. One model was generated for each dependent variable.

### 2.7. Statistical Analyses

All experiments and measurements were performed in triplicate and the data was analyzed by a randomized complete block design using the statistical software SPSS (v. 19). Duncan’s tests were used to perform mean comparisons and to determine the significance of the differences. In all cases, the criterion for statistical significance was *p* < 0.05.

## 3. Results

### 3.1. Non-Fermented Wheat Germ Sample Composition

The chemical composition of the original wheat germ biomass prior to fermentation was 14% of fat, 32% of protein, 15.5% of moisture, and 2.5% of ash.

### 3.2. Modelling the Fermentation Process of Wheat Germ

The matrix design and the experimental responses (TPC, DMBQ, and DPPH radical scavenging activity) for each run are presented in Table 1. There was considerable variation in the results obtained across the different responses analyzed, with ranges for TPC (1.59–3.99 mg GAE/g freeze-dried sample), DMBQ (0.06–0.64 mg DMBQ/g freeze-dried sample), and DPPH radical scavenging activity (50.01–89.15%). The highest yields TPC (3.99 mg GAE/g freeze-dried sample), DMBQ (0.64 mg DMBQ/g freeze-dried sample), and DPPH radical scavenging activity (88.95%) were achieved when wheat germ was fermented at pH of 6, during 48 h and using the bacteria (*L. plantarum*).

### 3.3. Modelling TPC during Fermentation

**Figure 1:**
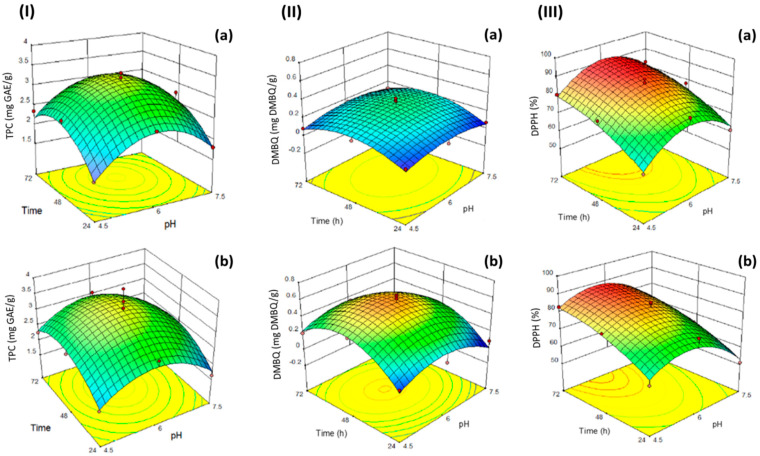
Contour plots (2D) and response surface plots (3D) of the effect of pH and fermentation times on the (**I**) TPC (mg gallic acid equivalents (GAE)/g freeze-dried sample), (**II**) DMBQ contents (mg DMBQ/g freeze-dried sample) and (**III**) DPPH (%) of wheat germ samples following a fermentation process using either: (**a**) yeast (*S*. *cerevisiae*) or (**b**) bacteria (*L. plantarum*).

Contour plots (2D) and response surface plots (3D) were generated from the previously described modeling equations for TPC as a function of different pH and fermentation times when using either bacteria or yeast (Figure 1).

Overall, the TPC of the fermented samples ranged between 1.59–3.99 mg GAE/g freeze-dried samples (Table 1). Keeping the pH constant and independent of the type of microorganism used for the fermentation, the TPC of the fermented wheat germ increased with an increased fermentation time, reaching its maximum levels at 48 h. Further increases in fermentation time resulted in unchanged or even slightly declined TPC levels. When the fermentation was performed using yeast, the highest TPC was 3.45 mg GAE/g freeze-dried sample, achieved at pH 6.0 and 48 h of fermentation time.

### 3.4. Modeling DMBQ Contents during Fermentation

The levels of DMBQ ranged from 0.06 to 0.64 mg DMBQ/g freeze-dried sample, depending on the fermentation conditions. Fermentation with either bacteria or yeasts contributed to increased levels of DMBQ, particularly at pH of 6.0 and fermentation time of 48 h, while further increases in any of these parameters resulted in reduced DMBQ contents.

### 3.5. Modelling DPPH Radical Scavenging Activity during Fermentation

The influence of the fermentation conditions on the DPPH radical scavenging activity of fermented wheat germ as a function of different pH and fermentation times when using either bacteria or yeast is shown in Figure 1. The levels of DPPH radical scavenging activity of fermented wheat germ ranged from 50.01 to 88.95%. Overall, when at the same pH values, the DPPH radical scavenging activity increased by increasing the fermentation time, achieving its highest value of 89.1% at a pH of 6.0 and fermentation time of 72 h. The process of fermentation significantly increased the DPPH radical scavenging activity of the samples compared to those of non-fermented wheat germ.

### 3.6. Optimum Conditions for Wheat Germ Fermentation

The coefficients provided in Table 2 indicate the effect of every independent parameter (pH (X_1_), time (X_2_), and type of microorganism (X_3_)) on the dependent variables. The magnitude of these coefficients relates to the weight of their effect, and the sign of the relationship (positive and negative) indicates an increase and decrease in the experimental responses, respectively. The results of the ANOVA indicated that the goodness-of-fit of quadratic polynomial models for all dependent variables was significant (*p* < 0.0001) (see Table 2). The mathematical models generated from the experimental data for TPC (Y_1_), DMBQ content (Y_2_), and DPPH (Y_3_) are expressed by the following equations:

(3) $$Y_{1}=3.29+0{.22X}_{2}-\;0{.75X}_{1}^{2}-\;0{.56X}_{2}^{2}$$

(4) $$Y_{2}=0.42+0{.052X}_{2}+0{.081X}_{3}-\;0{.13X}_{1}^{2}-\;0{.2X}_{2}^{2}\;$$

(5) $$Y_{3}=84.75\;-\;2{.35X}_{1}+8{.91X}_{2}+1{.74X}_{3}+3{.36X}_{1}X_{3}-\;11{.88X}_{1}^{2}-\;3{.61X}_{2}^{2}\;$$

The high values of R^2^ and adjusted R^2^ (>0.80 in all the cases) indicated that the suggested models work well to elucidate the relationship between the variables proposed. The CV values for all the dependent variables were also low (<10% in all cases), indicating that the variation in the mean value is low and the proposed model has sufficient precision and reliability. The adequate precision measures the signal-to-noise ratio, and a ratio > 4 was considered desirable [17]. The adequate precision values of the current models—10.79 for TPC, 10.091 for DMBQ, and 23.162 for DPPH—suggest that the fitted models have a very good signal-to-noise ratio. Furthermore, the lack-of-fit values were also non-significant for all response models of the current study. Based on the model equations provided by RSM for each of the optimization objectives defined, the independent variables were subsequently adjusted using the RSM package’s response optimizer. A numerical optimization was performed to predict the optimum levels of each of the independent variables to obtain maximum values of TPC, DMBQ, and DPPH radical scavenging activity. The corresponding optimum conditions were achieved by the bacterial fermentation of wheat germ at pH 6 during 48 h of fermentation with a high desirability coefficient of 0.89. The desirability lies between 0 and 1, and it represents the closeness of a response to its ideal value. Under these optimum conditions, the levels of TPC, DMBQ, and DPPH radical scavenging activity were 3.33 mg of GAE/g, 0.56 mg DMBQ/g and 86.49%, respectively.

### 3.7. Further Chemical and In Vitro Biological Activity of Optimally Fermented Wheat Germ

Fermented wheat samples generated using optimum fermentation conditions (bacteria, pH 6 and 48 h fermentation) were further analyzed for their contents of certain bioactive compounds (peptide and GABA contents) with proven health benefits frequently described when fermenting wheat germ. Moreover, the chemical composition of non-fermented wheat germ and yeast-fermented wheat germ (pH 6 and 48 h fermentation) are also reported for comparison purposes.

### 3.8. Peptide Contents of Optimally Fermented Wheat Germ

The peptide content of wheat germ in this study was 35.5 μg/mL, rising to levels of 607 μg/mL following an optimized fermentation process using bacteria, is also higher than those of wheat germ fermented using yeast following similar fermentation parameters of 532.50 μg/mL.

### 3.9. GABA Contents of Optimally Fermented Wheat Germ

**Figure 2:**
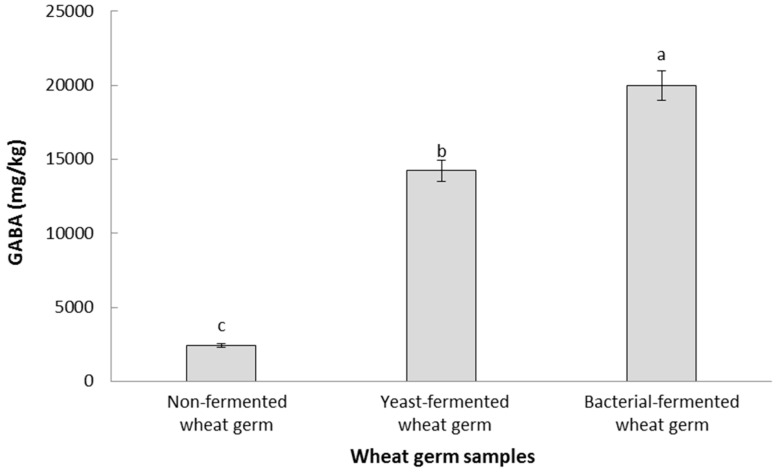
GABA contents (mg GABA/kg freeze-dried sample) in raw and fermented wheat germ samples. Data are expressed as average ± standard deviation. Different letters indicate statistical differences (*p* < 0.05) in the GABA contents between the different wheat germ samples.

Figure 2 summarizes the main changes in GABA contents between raw, yeast-fermented, and bacterial-fermented wheat germs. GABA content in non-fermented wheat germ samples was 2421.67 mg GABA/kg freeze-dried sample, increasing up to 13,675.62 mg/kg following yeast fermentation and achieving maximum levels following bacterial fermentation (19,983.88 mg/kg).

## 4. Discussion

The results of non-fermented wheat germ composition were in close agreement with those of Zhang, Xiao, Dong, Wu, Yao, and Zhou [2], who reported the protein content of wheat germ before fermentation with *L. plantarum* to be 32.9%. The TPC, DMBQ, and DPPH radical scavenging activity levels of non-fermented wheat germ in the current study were 0.77 mg of GAE/g, 0.12 mg/g and 23.22%, respectively.

This study showed great variations in the TPC of wheat germ after the samples were fermented under different processing conditions (see Table 1). Polyphenols, as a group of antioxidant molecules, play key roles in the prevention of several diseases, including cancer. Fermentation has been reported as an effective method to considerably enhance the content of polyphenols in the resulting products [18]. It is worth mentioning that the fermented wheat germ sample exhibited a significant enhancement in its TPC compared to its non-fermented counterpart, particularly when using the optimized conditions designed in this study to enhance the fermentation process. These results are in agreement with those of Zheng et al. [19], which achieved the highest phenolic contents in fermented wheat germ using *Saccharomyces cerevisiae* after 48 h of fermentation (3.6 mg GAE/g sample) that declined to 1.5 mg GAE/g sample when increasing the time of fermentation. TPC of wheat germ following a bacterial fermentation during 48 h was significantly higher than those described using yeast following the same experimental conditions. Similar results were also achieved by other researchers using various microorganism types during the fermentation of wheat germ. Liu, Chen, Shao, Wang, and Zhan [10] reported levels of TPC of 10.55 mg GAE/g of non-fermented wheat germ that increased up to 26.02 mg GAE/g following a 72 h fermentation process using Bacillus subtilis. Sandhu, Punia, and Kaur [18] also reported that the fungal fermentation of wheat germ with Aspergillus awamorinakazawa achieved increases in TPC from 1.3 mg GAE/g to 3.54 mg GAE/g after 2 days. LAB and *S. cerevisiae*, which are the focus of the current study, contain a wide range of enzymes—β-glucosidase, carboxylase, α-glucosidase, and phosphokinase—that are able to disrupt most of the fibers present in the wheat germ’s cell walls, such as cellulose, hemicellulose, and pentosans [3,15,20]. Thus, during the fermentation process, these enzymes will generate a breakage of the polyphenol–hemicellulose bonds which will ultimately lead to the increases in TPC also appreciated in this study.

DMBQ is a derivative of quinones that contribute greatly to the beneficial biological properties attributed to the consumption of wheat germ [19]. Overall, the results confirm that fermentation resulted in a significant increase of these beneficial compounds and thus, these fermented products can have an increased value when sold as nutraceuticals or functional foods, particularly when using the optimized fermentation conditions determined in this study. Zheng, Guo, Zhu, Peng, and Zhou [15] used a combined artificial neural network and genetic algorithm strategy to optimize wheat germ fermentation by the *Saccharomyces cerevisiae*, achieving a maximum content of quinones of 0.939 mg/g sample. Similarly, Zhang, Xiao, Dong, Wu, Yao, and Zhou [2] reported wheat germ contained approximately 33.8 µg DMBQ/g, and after fermentation with *Lactobacillus plantarum* dy-1, the concentration of DMBQ increased to 181.1 µg DMBQ/g. Rizzello, Mueller, Coda, Reipsch, Nionelli, Curiel, and Gobbetti [3] also demonstrated increases in DMBQ from 0.035 to 0.252 mg/g achieved by LAB fermentation. The mechanism of release of hydroquinones (which exist as β-glucosides) from wheat germ during fermentation is attributed to the action of β-glucosidase released during both yeast and bacterial fermentations. When these compounds are released via the breakage of β-glucosidic bonds, they are oxidized to DMBQ. Moreover, in wheat germ, high levels of β-glucosidase and peroxidase enzymes can be naturally present, contributing further to the formation of DMBQ [20].

Increased antioxidant activity of different metabolites has been linked to other biological properties also displayed by these compounds, including their anticarcinogenic activity [20]. Thus, during the process of optimization, antioxidant activities were used as a marker of in vitro biological properties of the fermented wheat germ. Liu, Chen, Shao, Wang, and Zhan [10] fermented wheat germ using *L. plantarum* and reported differences in the antioxidant activities (expressed as % DPPH radical scavenging activity) of samples at early stages of fermentation and those fermented after 72 h. The authors reported antioxidant activities of 10% in the raw wheat germ samples that increased to reach levels of approximately 78% when fermenting the products with *L. plantarum*. Rizzello, Nionelli, Coda, De Angelis and Gobbetti [4] also reported that the fermentation of wheat germ with *L. plantarum* LB1 and *L. rossiae* LB5 led to an enhancement of 33% in the antioxidant activities of fermented wheat germ. The improved antioxidant activity reported in multiple studies as a result of the fermentation process may be mainly related to the production of phenolic and flavonoid compounds [3] as well as to the release of peptides through microbial-derived hydrolysis during the process of fermentation [10].

Overall, the process of fermentation increased the release of bioactive peptides from wheat germ, especially when using the optimized protocol designed in this study. Bioactive peptides can be produced by enzymatic hydrolysis during the processes of fermentation, germination, and ripening [5], and they may have an active role in contributing to the antioxidant and anticarcinogenic activities of wheat germ. Liu, Chen, Shao, Wang, and Zhan [10] reported that the peptide contents of wheat germ increased from 4.31 to 29.68% during the first 48 h of fermentation with Bacillus subtilis, while these levels were reduced to 25.80% at 72 h. The authors attributed this increased peptide content to the activity of a proteinase secreted by Bacillus subtilis which could hydrolyze protein to several peptides. These findings were further supported by Niu et al. [21], who reported increased peptide content in wheat germ samples when fermented for less than 48 h, while the concentration of these compounds declined following additional fermentation time.

GABA is a four-carbon non-protein amino acid that is involved in multiple biological processes relevant to human health, including control of blood pressure, antidiabetic, anticarcinogenic, anti-obesity, and tranquilizing effects, which minimizes the risks of heart diseases and Alzheimer’s [22,23]. Thus, the significant increase in GABA contents also appreciated when fermenting wheat germ using the optimum protocol developed in this study also indicates the potential additional health benefits that could be achieved following the fermentation conditions explored in this study. Similar to the current study results, Rizzello, Nionelli, Coda, De Angelis, and Gobbetti [4] reported an increase in GABA contents from 903 mg/kg in raw samples to 2043 mg/kg when the samples were fermented by *Lactobacillus plantarum* LB1 and *Lactobacillus rossiae* LB5. The higher levels of GABA in the fermented samples of the current study can be attributed to the different bacterial strains used to optimize the fermentation of wheat germ.

## 5. Conclusions

The bacterial fermentation of wheat germ using *L. plantarum* was more efficient compared to yeast fermentation using *S. cerevisiae* for the generation of bioactive compounds and increased biological activities in vitro of fermented wheat germ. Moreover, the fermentation process using *L. plantarum* was also optimized for increased bioactive compounds and biological properties. Under optimum fermentation conditions (pH 6, 48 h), bacterial fermentation significantly improved the contents of TPC, DMBQ, and DPPH of fermented wheat germ at higher levels than those described in both the raw and yeast fermented wheat germ. Fermentation parameter modifications, such as increased fermentation time (72 h) or increases of pH beyond those optimum conditions, did not improve and, in some cases, even reduced the generation of the compounds analyzed and their biological properties in vitro. The insights gained into the understanding of the effects of different fermentation parameters of wheat germ by *L. plantarum* and *S. cerevisiae* can be potentially used at an industrial level by the food industry in order to achieve value-added products with specific functional properties, such as antioxidant properties, from wheat germ that is currently considered as a low-value by-product from the flour and milling industries.

## Figures and Tables

**Table 1:** Matrix of experimental design defined by central composite design for the optimization of the process of fermentation of wheat germ. The main experimental responses (total phenolic content (TPC), dimethoxy benzoquinone (DMBQ) and 1,1-diphenyl-2-picrylhydrazyl (DPPH) radical scavenging activity) are expressed as average ± standard deviation.

Experiment	pH	Time (h)	Type of Microorganism ^1^	TPC ^2^	DMBQ ^3^	DPPH ^4^
1	7.5	24	Yeast	1.80 ± 0.03	0.07 ± 0.01	50.01 ± 1.12
2	7.5	48	bacteria	2.43 ± 0.05	0.21 ± 0.01	78.13 ± 2.03
3	7.5	72	bacteria	2.05 ± 0.02	0.16 ± 0.03	82.37 ± 1.54
4	7.5	72	Yeast	2.33 ± 0.02	0.12 ± 0.02	71.01 ± 0.98
5	7.5	48	Yeast	2.76 ± 0.01	0.20 ± 0.01	65.09 ± 1.35
6	6.0	48	bacteria	2.89 ± 0.05	0.52 ± 0.03	65.82 ± 2.47
7	6.0	24	bacteria	2.75 ± 0.04	0.10 ± 0.04	77.78 ± 0.83
8	4.5	48	Yeast	2.53 ± 0.02	0.15 ± 0.03	77.53 ± 1.18
9	4.5	24	bacteria	1.83 ± 0.02	0.09 ± 0.02	59.49 ± 0.88
10	6.0	24	Yeast	2.46 ± 0.03	0.08 ± 0.02	75.44 ± 1.46
11	6.0	72	bacteria	3.05 ± 0.01	0.36 ± 0.01	89.15 ± 1.08
12	4.5	72	bacteria	2.26 ± 0.04	0.21 ± 0.03	80.19 ± 0.91
13	6.0	48	bacteria	2.95 ± 0.05	0.53 ± 0.01	83.24 ± 2.79
14	4.5	72	Yeast	2.33 ± 0.03	0.08 ± 0.03	82.77 ± 2.63
15	7.5	24	bacteria	1.64 ± 0.02	0.08 ± 0.03	61.08 ± 0.84
16	6.0	48	bacteria	3.41 ± 0.02	0.60 ± 0.04	85.64 ± 1.92
17	4.5	48	bacteria	2.54 ± 0.04	0.40 ± 0.03	75.85 ± 1.53
18	6.0	48	bacteria	3.62 ± 0.03	0.62 ± 0.02	86.88 ± 2.26
19	6.0	72	Yeast	2.75 ± 0.01	0.17 ± 0.02	87.27 ± 2.08
20	6.0	48	Yeast	2.90 ± 0.03	0.23 ± 0.01	80.19 ± 1.27
21	4.5	24	Yeast	1.59 ± 0.05	0.06 ± 0.01	61.08 ± 0.98
22	6.0	48	Yeast	3.45 ± 0.03	0.35 ± 0.03	85.00 ± 1.09
23	6.0	48	Yeast	3.10 ± 0.04	0.25 ± 0.04	81.80 ± 2.34
24	6.0	48	Yeast	3.20 ± 0.01	0.27 ± 0.02	82.34 ± 2.18
25	6.0	48	Yeast	3.34 ± 0.05	0.38 ± 0.02	85.73 ± 0.93
26	6.0	48	bacteria	3.99 ± 0.02	0.64 ± 0.03	88.95 ± 1.63

^1^ yeast (*Saccharomyces cerevisiae*) or bacteria (*Lactobacillus plantarum*); ^2^ TPC expressed in mg of gallic acid equivalent (GAE)/g freeze-dried sample; ^3^ DMBQ contents expressed in mg DMBQ/g freeze-dried sample; ^4^ DPPH radical scavenging activity expressed as %.

**Table 2:** Analysis of variance describing the effect of the different fermentation variables on the response variables (TPC, DMBQ, and DPPH) as linear, quadratic, and interactive terms and the coefficients for the predicted models.

Source	DF	TPC			DMBQ			DPPH		
		Coefficient	Sum of Squares	*p*-Value	Coefficient	Sum of Squares	*p*-Value	Coefficient	Sum of Squares	*p*-Value
Model	8	3.29	8.39	<0.0001 **	0.42	0.73	<0.0001 **	84.75	2439.02	<0.0001 **
X_1_	1	-	0.004	0.9418 ^ns^	-	0.001	0.6351 ^ns^	−2.35	66.40	0.0107 *
X_2_	1	0.22	0.61	0.0109 *	0.052	0.032	0.0435 *	8.91	951.83	<0.0001 **
X_3_	1	-	0.029	0.5402 ^ns^	0.081	0.17	0.0003 **	1.74	78.34	0.0063 **
X_1_X_2_	1	-	0.006	0.7694 ^ns^	-	0.002	0.9957 ^ns^	-	0.15	0.8927 ^ns^
X_1_X_3_	1	-	0.075	0.3291 ^ns^	-	0.009	0.3014 ^ns^	3.36	135.37	0.0008 **
X_2_X_3_	1	-	0.015	0.6625 ^ns^	-	0.007	0.3533 ^ns^	-	0.002	0.9869 ^ns^
X_1_^2^	1	−0.75	3.07	<0.0001 **	−0.13	0.098	0.0030 **	−11.88	779.06	<0.0001 **
X_2_^2^	1	−0.56	1.72	0.0002 **	−0.20	0.22	<0.0001 **	−3.61	72.12	0.0083 **
Residual	17		1.27			0.14			137.34	
Lack of Fit	9		0.23	0.9874 ^ns^		0.11	0.0725 ^ns^		89.04	0.2490 ^ns^
Pure Error	8		1.04			0.029			48.30	
Total	25		9.66			0.87			2576.36	
R^2^		0.8689			0.8390			0.9467		
Adj. R^2^		0.8072			0.8128			0.9216		
CV (%)		8.15			8.98			3.66		
Adequate precision		10.79			10.091			23.162		

** Highly significant (*p* < 0.01), * significant (*p* < 0.05) and ^ns^ for not significant.

## Data Availability

No new data were created or analyzed in this study. Data sharing is not applicable to this article.
